# Rapid antidepressant effects of the psychedelic ayahuasca in treatment-resistant depression: a randomized placebo-controlled trial

**DOI:** 10.1017/S0033291718001356

**Published:** 2019-03

**Authors:** Fernanda Palhano-Fontes, Dayanna Barreto, Heloisa Onias, Katia C. Andrade, Morgana M. Novaes, Jessica A. Pessoa, Sergio A. Mota-Rolim, Flávia L. Osório, Rafael Sanches, Rafael G. dos Santos, Luís Fernando Tófoli, Gabriela de Oliveira Silveira, Mauricio Yonamine, Jordi Riba, Francisco R. Santos, Antonio A. Silva-Junior, João C. Alchieri, Nicole L. Galvão-Coelho, Bruno Lobão-Soares, Jaime E. C. Hallak, Emerson Arcoverde, João P. Maia-de-Oliveira, Dráulio B. Araújo

**Affiliations:** 1 Brain Institute, Federal University of Rio Grande do Norte (UFRN), Natal/RN, Brazil; 2 Onofre Lopes University Hospital, UFRN, Natal/RN, Brazil; 3 Department of Clinical Medicine, UFRN, Natal/RN, Brazil; 4 Department of Neurosciences and Behaviour, University of São Paulo (USP), Ribeirão Preto/SP, Brazil; 5 National Institute of Science and Technology in Translational Medicine (INCT-TM), Ribeirão Preto/SP, Brazil; 6 Department of Medical Psychology and Psychiatry, University of Campinas, Campinas/SP, Brazil; 7 Department of Clinical Analysis and Toxicology, USP, São Paulo/SP, Brazil; 8 Sant Pau Institute of Biomedical Research, Barcelona, Spain; 9 Department of Pharmacy, UFRN, Natal/RN, – Brazil; 10 Department of Psychology, UFRN, Natal/RN, Brazil; 11 Department of Physiology, UFRN, Natal/RN, Brazil; 12 Department of Biophysics and Pharmacology, UFRN, Natal/RN, Brazil

**Keywords:** Ayahuasca, depression, HRS, MEQ, psychedelics, randomized controlled trial (RCT)

## Abstract

**Background:**

Recent open-label trials show that psychedelics, such as ayahuasca, hold promise as fast-onset antidepressants in treatment-resistant depression.

**Methods:**

To test the antidepressant effects of ayahuasca, we conducted a parallel-arm, double-blind randomized placebo-controlled trial in 29 patients with treatment-resistant depression. Patients received a single dose of either ayahuasca or placebo. We assessed changes in depression severity with the Montgomery-Åsberg Depression Rating Scale (MADRS) and the Hamilton Depression Rating scale at baseline, and at 1 (D1), 2 (D2), and 7 (D7) days after dosing.

**Results:**

We observed significant antidepressant effects of ayahuasca when compared with placebo at all-time points. MADRS scores were significantly lower in the ayahuasca group compared with placebo at D1 and D2 (*p* = 0.04), and at D7 (*p* < 0.0001). Between-group effect sizes increased from D1 to D7 (D1: Cohen's *d* = 0.84; D2: Cohen's *d* = 0.84; D7: Cohen's *d* = 1.49). Response rates were high for both groups at D1 and D2, and significantly higher in the ayahuasca group at D7 (64% *v.* 27%; *p* = 0.04). Remission rate showed a trend toward significance at D7 (36% *v.* 7%, *p* = 0.054).

**Conclusions:**

To our knowledge, this is the first controlled trial to test a psychedelic substance in treatment-resistant depression. Overall, this study brings new evidence supporting the safety and therapeutic value of ayahuasca, dosed within an appropriate setting, to help treat depression. This study is registered at http://clinicaltrials.gov (NCT02914769).

## Introduction

The World Health Organization estimates that more than 300 million people suffer from depression (World Health Organization, 2017), and about one-third do not respond to appropriate courses of at least three different antidepressants (Conway *et al.*, 2017). Most currently available antidepressants have a similar efficacy profile and mechanisms of action, based on the modulation of brain monoamines, and usually, take about 2 weeks to start being effective (Cai *et al.*, 2015; Conway *et al.*, 2017; Otte *et al.*, 2016).

Recent evidence, however, shows a rapid and significant antidepressant effect of ketamine, an N-methyl-D-aspartate (NMDA) antagonist frequently used in anesthesia. In recent randomized placebo-controlled trials with ketamine in treatment-resistant depression, the antidepressant effects peaked 1 day after dosing and remained significant for about 7 days (Berman *et al.*, 2000; Zarate *et al.*, 2006; Murrough *et al.*, 2013; Lapidus *et al.*, 2014).

Additionally, research with serotonergic psychedelics has gained momentum (Vollenweider and Kometer, 2010). A few centers around the world are currently exploring how these substances affect the brain, and also probing their potential in treating different psychiatric conditions, including mood disorders (Grob *et al.*, 2011; Osório *et al.*, 2015; Carhart-Harris *et al.*, 2016; Griffiths *et al.*, 2016; Ross *et al.*, 2016; Sanches *et al.*, 2016). For instance, recent open-label trials show that psychedelics, such as ayahuasca and psilocybin, hold promise as fast-onset antidepressants in treatment-resistant patients (Osório *et al.*, 2015; Carhart-Harris *et al.*, 2016; Sanches *et al.*, 2016).

Ayahuasca is a brew traditionally used for healing and spiritual purposes by indigenous populations of the Amazon Basin (Luna, 2011; Spruce and Wallace, 1908). In the 1930s, it began to be used in religious settings of Brazilian small urban centers, reaching large cities in the 1980s and expanding since then to several other parts of the world (Labate and Jungaberle, 2011). In Brazil, ayahuasca has a legal status for ritual use since 1987. Ayahuasca is most often prepared by decoction of two plants (McKenna *et al.*, 1984): *Psychotria viridis* that contains the psychedelic N,N-dimethyltryptamine (N,N-DMT), a serotonin and sigma-1 receptors agonist (Carbonaro and Gatch, 2016), and *Banisteriopsis caapi*, rich in reversible monoamine oxidase inhibitors (MAOi) such as harmine, harmaline, and tetrahydroharmine (Riba *et al.*, 2003).

The acute psychological effects of ayahuasca last around 4 h and include intense perceptual, cognitive, emotional, and affective changes (Shanon, 2002; Riba *et al.*, 2003; Frecska *et al.*, 2016). Although nausea, vomiting, and diarrhea are often reported, mounting evidence points to a positive safety profile of ayahuasca. For instance, ayahuasca is not addictive and has not been associated with psychopathological, personality, or cognitive deterioration, and it promotes only moderate sympathomimetic effects (Grob *et al.*, 1996; Callaway *et al.*, 1999; Dos Santos *et al.*, 2011; Bouso *et al.*, 2012; Barbosa *et al.*, 2016).

In a recent open-label trial, 17 patients with major depressive disorder attended a single dosing session with ayahuasca. Depression severity was assessed before, during and after dosing, using the Hamilton Depression Rating scale (HAM-D) and the Montgomery–Åsberg Depression Rating Scale (MADRS) (Sanches *et al.*, 2016). Significant reduction in depression severity was found already in the first hours after dosing, an effect that remained significant for 21 days (Osório *et al.*, 2015; Sanches *et al.*, 2016).

Although promising, these studies have not controlled for the placebo effect, which can be remarkably high in clinical trials for depression, reaching 30–40% of the patients (Sonawalla and Rosenbaum, 2002). To address this issue, and to further test the antidepressant effects of ayahuasca, we conducted a randomized placebo-controlled trial in patients with treatment-resistant depression. Additionally, we explored for correlations between the antidepressant and the acute effects of ayahuasca.

## Materials and methods

### Study design and participants

This study is a double-blind parallel-arm randomized placebo-controlled trial. Patients were recruited from psychiatrist referrals at local outpatient psychiatric units or through media advertisements. All procedures took place at the Onofre Lopes University Hospital (HUOL), Natal-RN, Brazil. The University Hospital Research Ethics Committee approved the study (# 579.479), and all subjects provided written informed consent before participation. This study is registered at http://clinicaltrials.gov (NCT02914769).

We recruited adults aged 18–60 years who met criteria for the unipolar major depressive disorder as diagnosed by the Structured Clinical Interview for Axis I (DSM-IV). Only treatment-resistant patients were selected, defined herein as those with inadequate responses to at least two antidepressant medications from different classes (Conway *et al.*, 2017). Selected patients were in a current moderate-to-severe depressive episode at screening (HAM-D⩾17). Patients were submitted to a full clinical evaluation by a trained psychiatrist that included anamneses, mental health evaluation, and screening for either personal or family history of mania or bipolar disorder. We adopted the following exclusion criteria: previous experience with ayahuasca, current medical disease based on history, pregnancy, current or previous history of neurological disorders, personal or family history of schizophrenia or bipolar affective disorder, personal or family history of mania or hypomania, use of substances of abuse, and suicidal risk.

### Randomization and masking

Patients were randomly assigned (1:1) to receive ayahuasca or placebo using permuted blocks of size 10. All investigators and patients were blind to intervention assignment, which was kept only in the database and with the pharmacy administrators. Masking was further achieved by ensuring that all patients were naïve to ayahuasca, and by randomly assigning, for each patient, different psychiatrists for the dosing session and for the follow-up assessments. Psychiatrists’ blindness was not assessed.

### Procedures

We used the MADRS and the HAM-D (Carneiro *et al.*, 2015) to access depression severity. MADRS assessments were at baseline (one day before dosing), and at 1 (D1), 2 (D2), and 7 (D7) days after dosing. HAM-D was applied only at baseline and D7, as it was designed to access depression symptoms present in the last week (Hamilton, 1960).

The liquid used as placebo was designed to simulate organoleptic properties (taste and color) of ayahuasca, such as a bitter and sour taste, and a brownish color. It contained water, yeast, citric acid, zinc sulfate and caramel colorant. The presence of zinc sulfate also produced low to modest gastrointestinal distress. A single ayahuasca batch was used throughout the study, which was prepared and provided free of charge by a branch of the Barquinha church based at Ji-Paraná-RO, Brazil.

To assess alkaloids concentrations and stability of the batch, samples of ayahuasca were quantified at two different time points by mass spectroscopy analysis. On average, the ayahuasca used contained (mean ± s.d.): 0.36 ± 0.01 mg/ml of N, N-DMT, 1.86 ± 0.11 mg/ml of harmine, 0.24 ± 0.03 mg/ml of harmaline, and 1.20 ± 0.05 mg/ml of tetrahydroharmine (online Supplementary Table S1).

After screening, patients underwent a washout period of 2 weeks on average and adjusted to the half-life time of the antidepressant medication in use. During dosing session, patients were not under any antidepressant medication, and a new treatment scheme was introduced only 7 days after dosing. If needed, benzodiazepines were allowed as a supporting hypnotic and/or anxiolytic agents (online Supplementary Table S2 for demographic and clinical characteristics).

Dosing sessions lasted approximately 8 h, from 8:00 a.m. to 4:00 p.m., and intake usually occurred at 10:00 a.m. After a light breakfast, patients were reminded about the effects they could experience, and strategies to help alleviating eventual difficulties. Patients were also told that they could receive ayahuasca and feel nothing, or placebo and feel something. Sessions took place in a quiet and comfortable living room-like environment, with a bed, a recliner, controlled temperature, natural, and dimmed light.

Patients received a single dose of 1 ml/kg of placebo or ayahuasca adjusted to contain 0.36 mg/kg of N, N-DMT. They were asked to remain quiet, with their eyes closed, while focusing on their body, thoughts, and emotions. They were also allowed to listen to a predefined music playlist. Patients received support throughout the session from at least two investigators who remained in a room next door, offering assistance when needed. Acute effects were assessed with the Clinician-Administered Dissociative States Scale (CADSS) (Bremner *et al.*, 1998), the Brief Psychiatric Rating Scale (BPRS) (Crippa *et al.*, 2001), and the Young Mania Rating Scale (YMRS) (Vilela *et al.*, 2005), applied at −10 min, +1:40 h, +2:40 h, and +4:00 h after intake.

When the acute psychedelic effects ceased, patients had a last psychiatric evaluation, debriefed their experience, and responded to the Hallucinogenic Rating Scale (HRS) (Strassman *et al.*, 1994) and Mystical Experience Questionnaire (MEQ30) (MacLean *et al.*, 2012). Around 4:00 p.m. they could go home accompanied by a relative or friend. Only four patients presenting a more delicate condition remained as inpatients in the hospital ward for an entire week. Patients were asked to return for follow-up assessments at 1, 2, and 7 days after dosing.

### Outcomes

The primary outcome measure was the change in depression severity assessed by the HAM-D scale, comparing baseline with seven days (D7) after dosing. The secondary outcome was the change in MADRS scores from baseline to 1 (D1), 2 (D2), and 7 (D7) days after dosing. We examined the proportion of patients meeting response criteria, defined as a reduction of 50% or more in baseline scores. Remission rates were also examined and were defined as HAM-D⩽7 or MADRS⩽10. We assessed response and remission rates using HAM-D (at D7) and MADRS (at D1, D2, and D7) scores. Safety and tolerability were assessed with the CADSS, the BPRS, and the YMRS applied during dosing session. We used the HRS and the MEQ30 to assess specific aspects of the psychedelic effects.

### Statistical analysis

Analyses adhered to a modified intent-to-treat principle, including all patients who completed assessments at baseline, dosing and D7. An estimated sample size of 42 patients was estimated in G*Power software to provide 80% power to detect a five-point HAM-D difference (standardized effect size = 0.9) between baseline and D7 with two-sided 5% significance. The initial estimation was based on our previous open-label trial with ayahuasca in treatment-resistant depression (Sanches *et al.*, 2016). A fixed-effects linear mixed model, with baseline scores as covariate, examined changes in HAM-D at D7, and MADRS at D1, D2, and D7. A Toeplitz covariance structure was the best fit to the data according to Akaike's information criterion. Missing data were estimated using restricted maximum-likelihood estimation. Main effects and treatment *v.* time interaction were evaluated. *Post-hoc t* tests were performed for between-groups comparisons at all time points, and Sidak's test was used to control for multiple comparisons. Cohen's *d* effect sizes were obtained for between and within group comparisons. Between-group effect sizes were calculated using the estimated means of each group at each time point. For within-group comparisons, effect sizes of each treatment were calculated separately, using the differences between a time point and baseline values. Differences in the proportion of responders/non-responders and remitters/non-remitters were estimated using Fisher's exact test. Odds ratio (OR) and number needed to treat (NNT) were also calculated. Data from patients whose HAM-D or MADRS scores were reduced by 50% or more between washout onset and baseline, or that were in remission at dosing, were not considered for statistical analysis. Fisher's exact test was used to assess differences in the proportion of adverse events between the two treatments. We used the Mann–Whitney test to evaluate between-group differences in BPRS+, CADSS, HRS, and MEQ30. We calculated Pearson correlations between changes in MADRS scores from baseline to D7, and the acute effects during dosing assessed by BPRS, CADSS, MEQ30, and HRS. Multiple comparisons correction was based on the number of factors of each scale (*N* = 6, for the HRS; *N* = 5, for the MEQ30). Significance was set at *p* < 0.05, two-tailed. We used IBM SPSS Statistic 20 and Prism 7 to run the analyses.

## Results

**Fig. 1.**
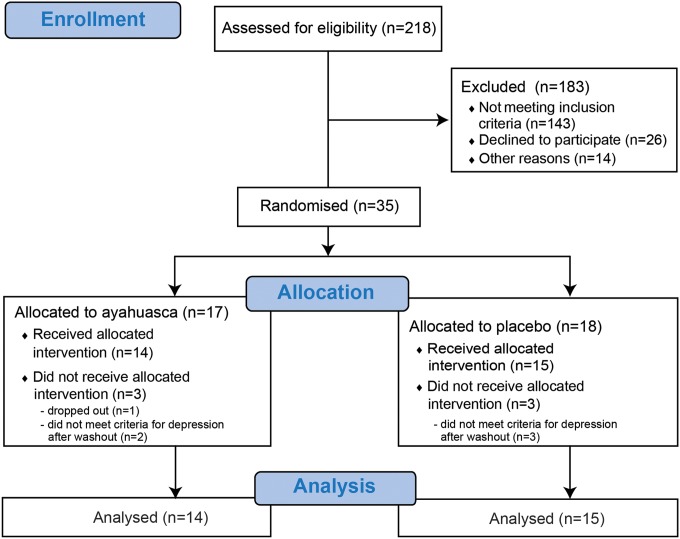
Trial profile.

From January 2014 to June 2016, we assessed 218 patients for eligibility, and 35 met criteria for the trial. Six subjects had to be excluded: five no longer met criteria for depression in the day of dosing, and one dropped out before dosing. Data from 29 patients were included in the analysis: 14 in the ayahuasca group and 15 in the placebo group. Figure 1 shows the trial profile.

On average, patients met criteria for moderate-to-severe depression (mean ± s.d.): HAM-D = 21.83 ± 5.35; MADRS = 33.03 ± 6.49. They had been experiencing depressive symptoms for 11.03 ± 9.70 years and had tried 3.86 ± 1.66 different previous unsuccessful antidepressants. Two patients had a previous history of electroconvulsive therapy. Most patients (76%) had a comorbid personality disorder, and 31% had a comorbid anxiety disorder. All patients were under regular use of benzodiazepines during the trial (online Supplementary Table S2 for clinical and demographics).

Demographic and clinical characteristics are summarized in Table 1 (online Supplementary Table S2). All patients were Brazilian, most female (72%), adults (42.03 ± 11.66 yo) from low socioeconomic status backgrounds: low educated (41% with <8 years of formal education) and living in low household income (41% earn <2 minimum wages).

**Table 1.** Sociodemographic & clinical characteristics

	Ayahuasca	Placebo
Participants, *n*	14	15
Age (years)	39.71 ± 11.26	44.2 ± 11.98
Gender (M/F)	3/11	5/10
Unemployed (%)	7/14 (50)	8/15 (53)
Household income		
<2 minimum wages (%)	6/14 (43)	6/15 (40)
2–5 wages (%)	4/14 (28)	7/15 (47)
6–10 wages (%)	1/14 (7)	1/15 (6.6)
11 or more wages (%)	3/14 (21)	1/15 (6.6)
Education		
Up to 8 years, *n* (%)	6/14 (43)	6/15 (40)
9–11 years, *n* (%)	3/14 (21)	5/15 (33)
12–16 years, *n* (%)	2/14 (14)	2/15 (13)
17 or more years, *n* (%)	3/14 (21)	2/15 (13)
Religion (%)		
Catholic	7/14 (50)	5/15 (33)
Protestant	4/14 (28)	1/15 (6.6)
Other	0/14 (0)	4/15 (27)
No religion	3/14 (21)	5/15 (33)
Ethnicity (%)		
Caucasian	9/14 (64)	8/15 (54)
Black	1/14 (7)	0/15 (0)
Pardo	4/14 (28)	7/15 (47)
Clinical characteristics		
Age of depression onset (years)	30.93 ± 10.19	30.87 ± 13.39
Illness duration (years)	8.78 ± 6.25	13.13 ± 11.92
Number of previous episodes	2.71 ± 1.32	3.53 ± 1.76
Length of current episode (months)	14.71 ± 18.92	10.13 ± 9.15
Failed antidepressant medications	3.93 ± 1.44	3.8 ± 1.89
History of ECT (%)	1/14 (7)	1/15 (6.6)
History of psychotherapy (%)	11/14 (79)	12/15 (80)
Anxiety disorder (%)	5/14 (36)	5/15 (33)
Personality disorder (%)	10/14 (71)	12/15 (80)
Melancholic (%)	12/14 (83)	12/15 (80)
Atypical (%)	2/14 (14)	3/15 (20)
Baseline HAM-D	24.07 ± 5.34	19.73 ± 4.59
Baseline MADRS	36.14 ± 6.12	30.13 ± 5.55

M, male; F, female; ECT, electroconvulsive therapy.

Values are (mean ± s.d.).

**Fig. 2.**
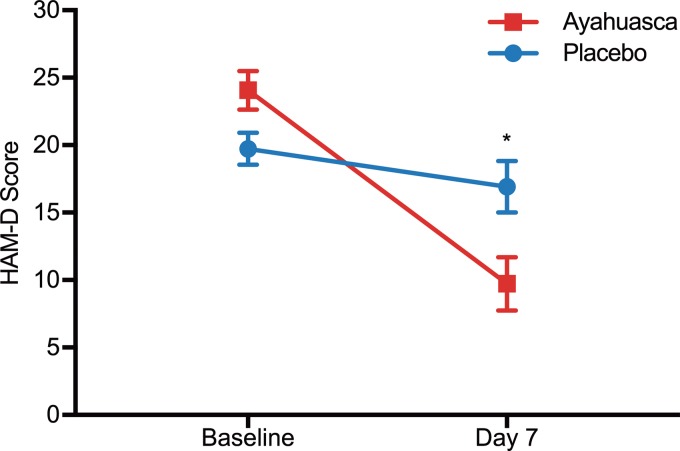
HAM-D scores at baseline and seven days after dosing. Statistical analysis shows a significant difference between ayahuasca (squares) and placebo (circles) seven days after dosing (*p* = 0.019). Between-group effect size is high (Cohen's *d* = 0.98). Values are (mean ± s.e.m.). HAM-D scores: mild depression (8–16), moderate (17–23), severe (⩾24).

Figure 2 shows changes in HAM-D scores from baseline to 7 days after dosing. We observed a significant between-groups difference at D7 (*F*_1_ = 6.31; *p* = 0.019), and patients treated with ayahuasca showed significantly reduced severity when compared with patients treated with placebo (online Supplementary Fig. S1 for individual HAM-D scores). Between-group effect size was large at D7 (Cohen's *d* = 0.98; 95% CI 0.21–1.75). Within-group effect size (online Supplementary Table S3) was large for the ayahuasca group (Cohen's *d* = 2.22; 95% CI 1.28–3.17), and medium for the placebo group (Cohen's *d* = 0.46; 95% CI −0.27 to 1.18).

**Fig. 3.**
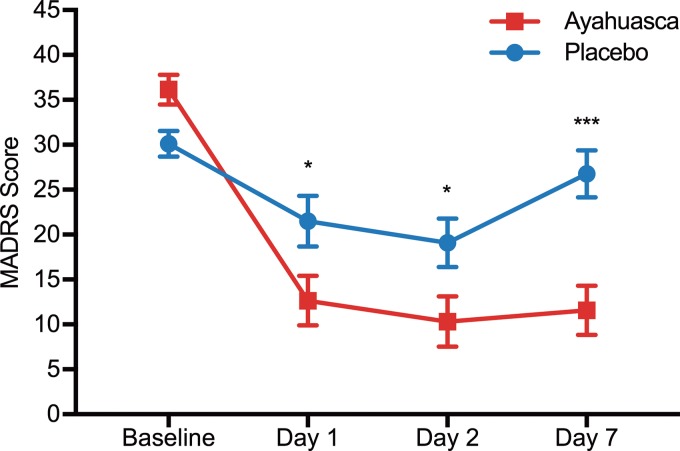
MADRS scores as a function of time. Significant differences are observed between ayahuasca (squares) and placebo (circles) at D1 (*p* = 0.04), D2 (*p* = 0.04) and D7 (*p* < 0.0001). Between groups effect sizes are high at all time points after dosing: D1 (Cohen's *d* = 0.84), D2 (Cohen's *d* = 0.84), and D7 (Cohen's *d* = 1.49). Values are (mean ± s.e.m.). MADRS scores: mild depression (11–19), moderate (20–34), severe (⩾35). *p < 0.05; ***p < 0.0001.

Figure 3 shows mean MADRS scores as a function of time. Linear mixed model showed a significant effect for time (*F*_2,34.4_ = 3.96; *p* = 0.028), treatment (*F*_1,27.7_ = 10.52; *p* = 0.003), but no treatment *v.* time interaction (*F*_2,34.4_ = 1.77; *p* = 0.185). We observed significant decreased depression severity already 1 day after dosing with ayahuasca compared with placebo (*F*_1,49.7_ = 4.58; *p* = 0.04). Depression severity persisted lower in the ayahuasca group at both D2 (*F*_1,50.3_ = 4.67; *p* = 0.04) and D7 (*F*_1,47_ = 14.81; *p* < 0.0001).

Between-groups effect size was large at D1 (Cohen's *d* = 0.84; 95% CI 0.05–1.62) and D2 (Cohen's *d* = 0.84; 95% CI 0.05–1.63) and largest at D7 (Cohen's *d* = 1.49; 95% CI 0.67–2.32). Within-group effect sizes (online Supplementary Table S4) were large for the ayahuasca at all time points: Cohen's *d* = 2.78 at D1 (95% CI 1.74–3.82), *d* = 3.05 at D2 (95% CI 1.94–4.16), and *d* = 2.90 at D7 (95% CI 1.84–3.97).

HAM-D response rate was significantly different between-groups at D7, with 57% of responders in the ayahuasca group against 20% in the placebo group [OR 5.33 (95% CI 1.11–22.58); *p* = 0.04; NNT = 2.69]. HAM-D remission rate showed a trend toward significance at D7: 43% in ayahuasca *v.* 13% in placebo [OR 4.87 (95% CI 0.77–26.73); *p* = 0.07; NNT = 3.39].

**Fig. 4.**
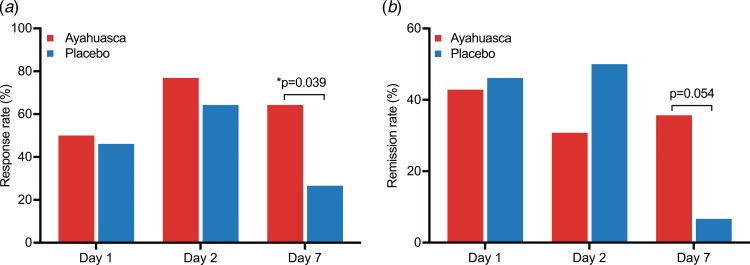
Response and remission rates as a function of time. Response (*a*) and remission (*b*) rates were high for both groups at D1 and D2. At D7, response rate was significantly higher for ayahuasca [OR 4.95 (95% CI 1.11–21.02); *p* = 0.04; NNT = 2.66], while remission rate showed a trend toward significance [OR 7.78 (95% CI 0.81–77.48); *p* = 0.054; NNT = 3.44].

Figure 4*a* shows MADRS response rates as a function of time. At D1, response rates were high for both groups: 50% in the ayahuasca group, and 46% in the placebo group [OR 1.17 (95% CI 0.26–5.48); *p* = 0.87; NNT = 26]. At D2, they remained high in both groups: 77% in the ayahuasca group and 64% in the placebo [OR 1.85 (95% CI 0.29–8.40); *p* = 0.43; NNT = 7.91]. Response rate was statistically different at D7: 64% of responders in the ayahuasca group, and 27% in the placebo [OR 4.95 (95% CI 1.11–21.02); *p* = 0.04; NNT = 2.66]. Figure 4*b* shows the MADRS remission rates as a function of time. At D1, the remission rate was of 42% in the ayahuasca group and 46% in the placebo group (*p* = 0.86), at D2, 31% in the ayahuasca group and 50% in the placebo group (*p* = 0.31). At D7 MADRS remission rate showed a trend toward significance: 36% of remitters in the ayahuasca group and 7% in the placebo [OR 7.78 (95% CI 0.81–77.48); *p* = 0.054; NNT = 3.44].

Online Supplementary Figs. S1 and S2 show individual MADRS scores %-changes from baseline, at all time points. Although individual variance was high, we found improvement in depression severity for all patients in the ayahuasca group 7 days after dosing, while four patients in the placebo group have worsened their symptoms.

Patients exhibited transient acute changes in CADSS and BPRS+ scales, with slightly increased scores at +1:40 h after ayahuasca intake: 34.8% (BPRS+) and 21.6% (CADSS) (online Supplementary Table S5). There was a trend increased toward significance observed in CADSS scores at 1:40 h after ayahuasca intake (*p* = 0.052). There were no significant changes in BPRS+ scores at any time point (online Supplementary Table S5). Changes in BPRS+ and CADSS scores did not correlate with improvements in depression symptoms (online Supplementary Fig. S3). We did not observe significant increased manic symptoms as measured by the YMRS at 1:40 h after ayahuasca intake (*p* = 0.26). We also observed transient nausea (Aya = 71%, Pla = 26%; *p* = 0.027), vomiting (Aya = 57%, Pla = 0%; *p* = 0.0007), transient anxiety (Aya = 50%, Pla = 73%; *p* = 0.263), restlessness (Aya = 50%, Pla = 20%; *p* = 0.128), and transient headache (Aya = 42%, Pla = 53%; *p* = 0.715) (online Supplementary Table S6).

The average and standard error of mean (s.e.m.) for each factor of both scales (HRS and MEQ30) are presented in online Supplementary Table S7, and in online Supplementary Figs. S4 and S5. Two patients did not respond to the HRS, leaving 27 respondents: 13 in the ayahuasca group, 14 in the placebo. The MEQ30 was added to the study with the trial already ongoing, and only 15 patients responded to it: eight in the ayahuasca group, seven in the placebo.

**Fig. 5.**
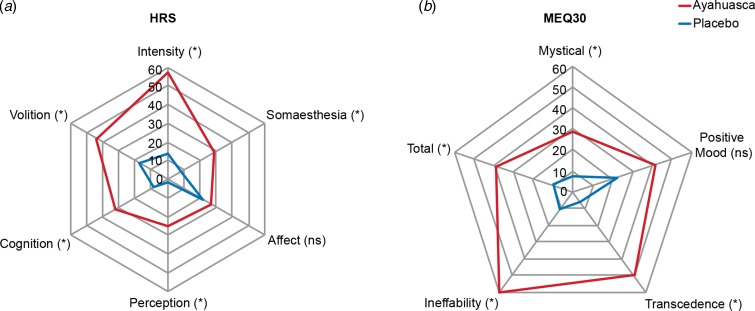
HRS subscales and MEQ30 factors during the dosing session. (*a*) Significantly higher scores in the ayahuasca group in five HRS subscales: perception (*p* < 0.0001), somaesthesia (*p* < 0.0001), cognition (*p* < 0.0001), intensity (*p* < 0.0001), and volition (*p* = 0.0003). Only affect was not significantly different between groups (*p* = 0.38). (*b*) Significantly higher MEQ30 scores in the ayahuasca group in the total MEQ30 score (*p* = 0.004), and three of its factors: mystical (*p* = 0.049), transcendence of time and space (*p* = 0.0008), and ineffability (*p* = 0.003), except for the positive mood (*p* = 0.32). Values are expressed as a percentage of maximum possible score.

Figure 5*a* shows the HRS average score for all six subscales. We found significant differences between groups in five of them. The ayahuasca group scored higher than the placebo group in perception (*p* < 0.0001), somaesthesia (*p* < 0.0001), cognition (*p* < 0.0001), intensity (*p* < 0.0001), and volition (*p* = 0.0003). Only affect was not significantly different between groups (*p* = 0.38). Figure 5*b* shows scores for all factors of the MEQ30. We found significant between-groups differences in mystical (*p* = 0.049), transcendence of time and space (*p* = 0.0008), ineffability (*p* = 0.003), and in total MEQ score (*p* = 0.004). For all of these, the ayahuasca group scored higher than the placebo group. The only positive mood was not significantly different between groups (*p* = 0.32).

Correlations between HRS and MADRS changes from baseline to D7 were not statistically significant when assessing each group separately, ayahuasca, or placebo (online Supplementary Fig. S6). However, we observed a positive significant correlation between MADRS changes at D7 with the HRS subscale ‘perception’ (*r* = 0.90, *p* = 0.002), when considering the subgroup of ayahuasca responders only (online Supplementary Fig. S7).

Despite the small number of patients, we found a negative correlation between changes in MADRS scores and the MEQ30 factor transcendence of time and space in patients in the ayahuasca group (*r* = −0.84, *p* = 0.009). The remaining three factors (ineffability, mystical, and positive mood) and MEQ30 total score were not significantly correlated with MADRS score changes (online Supplementary Fig. S8).

## Discussion

We found evidence of rapid antidepressant effect after a single dosing session with ayahuasca when compared with placebo. Depression severity changed significantly but differently for the ayahuasca and placebo groups. Improvements in the psychiatric scales in the ayahuasca group were significantly higher than those of the placebo group at all time points after dosing, with increasing between-group effect sizes from D1 to D7. Response rates were high for both groups at D1 and D2 and were significantly higher in the ayahuasca group at D7. Between-groups remission rate showed a trend toward significance at D7.

The within-group effect size found for ayahuasca at D7 (Cohen's *d* = 2.22) is compatible with our earlier open-label study (Cohen's *d* at D7 = 1.83) (Sanches *et al.*, 2016), and compatible with the one found in a recent open-label trial with psilocybin for depression (Hedges’ *g* = 3.1) (Carhart-Harris *et al.*, 2016). Our results are comparable with randomized controlled trials that used ketamine in treatment-resistant depression. Although both ketamine and ayahuasca are associated with rapid antidepressant effects, their response time-courses and mechanisms of action seem to differ. Previous studies with ketamine have found the largest between-group effect size at D1 (Cohen's *d* = 0.89), reducing toward D7 (Cohen's *d* = 0.41) (Berman *et al.*, 2000; Zarate *et al.*, 2006; Murrough *et al.*, 2013; Lapidus *et al.*, 2014). In contrast, the effect sizes observed herein were large, but smallest, at D1 (Cohen's *d* = 0.84), and largest at D7 (Cohen's *d* = 1.49). These differences are also reflected in the response rates. At D1, the response rate to ketamine lies between 37 and 70%, whereas in our study 50% of the patients responded to ayahuasca. At D7, the ketamine response rate ranges between 7 and 35% (Berman *et al.*, 2000; Zarate *et al.*, 2006; Murrough *et al.*, 2013; Lapidus *et al.*, 2014), while in our study 64% responded to ayahuasca.

The placebo effect was high in our study, and higher than most studies with. While we find a response rate to placebo of 46% at D1, and 26% at D7, ketamine trials have found a placebo effect of the order of 0–6% at D1, and 0–11% at D7 (Berman *et al.*, 2000; Zarate *et al.*, 2006; Murrough *et al.*, 2013; Lapidus *et al.*, 2014). Several factors may account for the high placebo effect observed herein. First, the higher placebo effect has been found in patients with low socioeconomic status (Sonawalla and Rosenbaum, 2002), which was the case of our study. Most patients were living under significant psychosocial stressors, and during our trial, they stayed at a ‘very comfortable and very supportive environment’, as reported by the patients themselves. Therefore, part of the increased placebo effects found in our study might be due to this ‘care effect’. Second, patients with comorbid personality disorders present higher placebo responses (Ripoll, 2013), and in our study, most patients (76%) also suffered from personality disorders, most of them in cluster B.

A growing body of evidence gives support to the observed rapid antidepressant effects of ayahuasca (Palhano-Fontes *et al.*, 2014). For instance, sigma-1 receptors (*σ*1R) have been implicated in depression, and it was reported to be activated by N, N-DMT (Cai *et al.*, 2015; Carbonaro and Gatch, 2016). Moreover, it has been shown that the administration of *σ*1R agonists results in antidepressant-like effects, which are blocked by *σ*1R antagonism (Cai *et al.*, 2015). Furthermore, *σ*1R upregulates neurotrophic factors such as brain-derived neurotrophic factor (BDNF) and nerve growth factor (NGF), proteins whose regulation and expression seem to be involved in the pathophysiology of depression (Cai *et al.*, 2015). Nevertheless, it is worth mentioning that antidepressants with σ1R agonist profile do not present clinically significant antidepressant effect. For instance, the antidepressant fluvoxamine, which has a high affinity for *σ*1R do not present response rates compatible to that was found herein (Delgado *et al.*, 1988; Hashimoto, 2009).

The effects observed might be in part due to the presence of MAOi in the brew. In fact, studies in animal models reported that chronic administration of harmine reduces immobility time, increases climbing and swimming time, reverses anhedonia, increases adrenal gland weight, and increases BDNF levels in the hippocampus (Fortunato *et al.*, 2010*a*, 2010*b*). All of these are compatible with antidepressant effects. Likewise, harmine seems to stimulate neurogenesis of human neural progenitor cells, derived from pluripotent stem cells (Dakic *et al.*, 2016), and progenitor cells from adult mice brains (Morales-García *et al.*, 2017), a mechanism also observed in rodents following antidepressant treatment. In addition, a recent study in rodents found that a single ayahuasca dose increases swimming time in a forced-swim test (Pic-Taylor *et al.*, 2015).

Brain circuits modulated by psychedelics show great overlap with those involved in mood disorders (Vollenweider and Kometer, 2010). We recently found that a single ayahuasca session in patients with depression increases blood flow in brain regions consistently implicated in the regulation of mood and emotions, such as the left nucleus accumbens, right insula and left subgenual area (Otte *et al.*, 2016; Sanches *et al.*, 2016). Moreover, we have shown that ayahuasca reduces the activity of the Default Mode Network (Palhano-Fontes *et al.*, 2015), a brain network found to be hyperactive in depression (Sheline *et al.*, 2009).

Over the last two decades, mental health evaluations of regular ayahuasca consumers have shown preserved cognitive function, increased well-being, reduction of anxiety, and depressive symptoms when compared to non-ayahuasca consumers (Grob *et al.*, 1996; Bouso *et al.*, 2012; Barbosa *et al.*, 2016). Moreover, a recent study observed that a single dose of ayahuasca enhanced mindfulness-related capacities (Soler *et al.*, 2016), and meditation practices have been associated with antidepressant effects (Segal *et al.*, 2010).

Prior studies suggest that elements of the psychedelic experience, such as experiences of mystical-type, account for the therapeutic benefit (Bogenschutz *et al.*, 2015; Garcia-Romeu *et al.*, 2015; Majić *et al.*, 2015; Griffiths *et al.*, 2016; Ross *et al.*, 2016). We found significant increased MEQ30 scores during the effects of ayahuasca. We also observed an inverse correlation between MADRS score changes at D7 with ‘transcendence of time and space’ MEQ30 factor.

Furthermore, HRS dimensions seem important to the clinical outcome, particularly ‘perception’, a subscale that comprehends changes in visual, auditory, and body sensations. Visions are common during the effects of ayahuasca, and are most frequent with the eyes closed (Shanon, 2002; De Araujo *et al.*, 2012). It has been suggested that visions may play an important role in the therapeutic effect of ayahuasca, as they may help bringing clarity to introspective events (Frecska *et al.*, 2016). It is interesting to observe that changes in perception taken alone are not sufficient to predict the positive clinical outcome, as for instance, we find that some patients presented increased scores in ‘perception’ without significant clinical response.

No serious adverse events were observed during or after dosing. Although 100% of the patients reported feeling safe, the ayahuasca session was not necessarily a pleasant experience. In fact, some patients reported the opposite, as the experience was accompanied by much psychological distress. Most patients reported nausea, and about 57% have vomited, although vomiting is traditionally not considered a side effect of ayahuasca, but rather part of a purging process (Tafur, 2017).

Although promising, this study has some caveats and limitations worth mentioning. The number of participants is modest, and therefore randomized trials in larger populations are necessary. The study was limited to patients with treatment-resistant depression, with a long course of illness, and high comorbid personality disorder, which altogether precludes a simple extension of these results to other classes of depression. Another challenge of the research with psychedelics is maintaining double blindness, as the effects of psychedelics are unique. We were particularly keen to ensure blindness throughout the entire experiment, and to that end, we adopted a series of additional measures to preserve blindness. All patients were naïve to ayahuasca, with no previous experience with any other psychedelic substance. Clinical evaluations involved a team of five psychiatrists. For every patient, one psychiatrist was responsible for clinical evaluation during the dosing session and a different one for the follow-up assessments. The substance used as placebo increased anxiety and induced nausea. In fact, five patients misclassified placebo as ayahuasca, and two of them showed a response at D7 (online Supplementary Table S2). Therefore, we believe blindness was adequately preserved in our study.

Since the prohibition of psychedelics in the late 1960s, research with these substances has almost come to a halt. Before research restrictions, psychedelics were at early stage testing for many psychiatric conditions, including obsessive-compulsive disorder and alcohol dependence. By mid-1960s, over 40.000 subjects had participated in clinical research with psychedelics, most of them in uncontrolled settings (Vollenweider and Kometer, 2010). To our knowledge, this is the first randomized placebo-controlled trial to investigate the antidepressant potential of a psychedelic in a population of patients with treatment-resistant depression. Overall, this study brings new evidence supporting the safety and therapeutic value of psychedelics, dosed within an appropriate setting, to help treat depression.

## Supplementary material

For supplementary material accompanying this paper visit https://doi.org/10.1017/S0033291718001356. click here to view supplementary material
