# Supplementary material for: Rapid antidepressant effects of the psychedelic ayahuasca in treatment-resistant depression: a randomized placebo-controlled trial
Source: Psychol Med. 2019 Mar;49(4):655–63. doi: 10.1017/S0033291718001356 (PMC6378413; doi:10.1017/S0033291718001356)
Supplement: Supplementary file 1 [file S0033291718001356sup001.docx]

**"Rapid antidepressant effects of the psychedelic ayahuasca in treatment-resistant depression: a randomized placebo-controlled trial."**

**Palhano-Fontes, *et al.*, 2018.**

**Supplementary material**

**Quantification of ayahuasca Alkaloids**

Ayahuasca sample extraction. Ayahuasca alkaloids extraction was performed according to the procedure described in Pires *et al.* (2009). First, 50 µL of sample solution was diluted with deionised water (1:100). Borate buffer 0.25 M pH 9.0 (3 mL) was added into 500 µL of the diluted sample solution and the internal standard diphenhydramine (100 μL of a solution of 10 µg/mL) was loaded onto a C18 cartridge previously conditioned (methanol 2.0 mL, deionised water 1.0 mL and borate buffer 2.0 mL). The loaded cartridge was further washed with deionised water (1.0 mL) and acetonitrile 10% (1.0mL). After drying the cartridges for 7 min, the analytes were eluted with methanol (2.0 mL). Of this solution, 2 μL was injected in the GC-NPD system.

Reagents and chemicals. Hydrogen borate, methanol and acetonitrile were purchased from Merck (Darmstadt, Germany). Classic Sep-Pack® C18 cartridges (360 mg) were purchased from Waters Co. (Bellefonte, PA, USA). N,N-Dimethyltryptamine was obtained from Cerilliant Corporation (Round Rock, Texas, EUA). Diphenhydramine, harmine hydrochloride and harmaline hydrochloride were purchased from Sigma Co. (St Louis, MO, USA). Tetrahydroharmine was synthesized according to the procedure described in Callaway et al. (1996).

GC-NPD analyses. Analyses for N,N-Dimethyltryptamine (N,N-DMT), harmine (HRM), harmaline (HRL) and tetrahydroharmine (THH) were performed using an Agilent gas chromatograph model 6890 equipped with a nitrogen–phosphorous detector and 7683 series automatic injector (Little Falls, DE, USA). Chromatographic separation was achieved on an HP Ultra-2 fused-silica capillary column (25 m × 0.2mm× 0.33 μm film thickness) using ultra-pure- grade nitrogen as carrier gas at 1.0 mL/min in a constant flow rate mode. Injections (2 μL) were made in splitless mode. The injector port and detector temperature was 280°C. The oven temperature was maintained at 70°C for 1 min; programmed at 30°C/min to 120°C, and 20°C/min to 300°C with a hold at 300°C for 4 min. The analytes were identified based on comparison of its relative retention time with the corresponding values of the internal standard diphenhydramine assayed in the same run. Quantification was based upon the ratio of the integrated peak area to the internal standard. The result was multiplied by 100 to compensate for dilution.

Alkaloid concentration. The ayahuasca batch was kept on a refrigerator in amber glass bottles. Two samples of ayahuasca were sent to GC-NPD analyses, at two different moments of the experiment, 11 months apart. Table S1 shows the two separate alkaloid concentration measurements.

**Table S1.** Alkaloid concentrations measured at two different moments of the experiment.

|  | May-15 | Apr-16 |
| --- | --- | --- |
| N,N-DMT (mg/ML) | 0.35 | 0.37 |
| THH (mg/ML) | 1.24 | 1.15 |
| HRL (mg/ML) | 0.26 | 0.21 |
| HRM (mg/ML) | 1.75 | 1.97 |

**Table S2.** Demographic and clinical characteristics.

|  | Sex | Age | Education | Employment status | Personality Disorder | Age at onset, y | Estimated illness duration, y | Number of previous episodes | Current episode, months | Past unsuccessful medications | Past psychotherapy | Past ECT #sessions | Benzodiazepine medications | Treatment classification |
| --- | --- | --- | --- | --- | --- | --- | --- | --- | --- | --- | --- | --- | --- | --- |
|  |  |  |  |  |  |  |  |  |  |  |  |  |  |  |
| A1 | F | 36 | Inc. elem. education | Sick leave | Histrionic | 26 | 10 | 4 | 3 | **SSRI**(2), NDRI, SNRI | Yes | None | CZP | Aya |
| A2 | F | 54 | Inc. elem. education | Unemployed | Histrionic | 33 | 21 | 3 | 3 | **SSRI**(3), TCA | Yes | None | CZP | - |
| A3 | F | 34 | Undergraduate | Employed | Borderline | 28 | 6 | 4 | 2 | **SSRI**(2), **NDRI** | Yes | None | CZP | Aya |
| A4 | M | 52 | Inc. elem. education | Unemployed | Histrionic + Dependent | 38 | 14 | 2 | 2 | TCA(2), **SSRI**(2) | Yes | None | DZP | Aya |
| A5 | F | 56 | Secondary education | Unemployed | Cluster B (undefined) | 53 | 3 | 1 | 36 | **SSRI**(2), **NDRI** | None | None | DZP | Aya |
| A6 | F | 47 | Postgraduate | Employed | None | 35 | 12 | 2 | 5 | **SSRI**(2), SNRI | None | None | CZP | Aya |
| A7 | M | 22 | Inc. undergraduate | Studying | Narcissistic + Borderline | 17 | 5 | 1 | 60 | SNRI(2), NDRI, SSRI, **TCA** | Yes | 16 | CZP | Aya |
| A8 | F | 45 | Inc. elem. education | Unemployed | Histrionic | 29 | 16 | 3 | 12 | TCA(2), **SSRI**(2) | Yes | None | CZP | Aya |
| A9 | M | 19 | Secondary education | Studying | None | 18 | 1 | 2 | 4 | **SSRI**, TCA | Yes | None | CZP | Aya |
| A10 | F | 39 | Secondary education | Unemployed | None | 38 | 1 | 2 | 6 | SSRI(2), **SARI**, TCA, **SNRI** | Yes | None | CZP | Aya |
| A11 | F | 47 | Postgraduate | Unemployed | Borderline | 45 | 2 | 3 | 4 | **TCA**, **SSRI**(2) | Yes | None | CZP | Aya |
| A12 | F | 33 | Postgraduate | Employed | None | 21 | 12 | 2 | 12 | **SSRI**(3), SNRI(2), **TCA**, MA, **NDRI** | Yes | None | CZP | Aya |
| A13 | F | 32 | Inc. elem. education | Sick leave | Borderline | 26 | 6 | 6 | 9 | TCA(2), SSRI(2)* | Yes | None | BZP | Aya |
| A14 | F | 40 | Elementary education | Unemployed | Borderline | 26 | 14 | 3 | 48 | SSRI(2), **SNRI** | None | None | CZP | Aya |
| P1 | M | 27 | Secondary education | Employed | Schizoid | 21 | 6 | 2 | 1 | **TCA**(2), SSRI(2), NDRI, **SNRI** | Yes | None | AZP | Pla |
| P2 | F | 50 | Secondary education | Unemployed | Histrionic | 41 | 9 | 4 | 8 | **SSRI**(2), NSSRI | Yes | None | DZP | Aya |
| P3 | F | 52 | Postgraduate | Unemployed | Borderline | 36 | 16 | 3 | 6 | **SNRI**, SSRI, TCA | Yes | 21 | APZ | Aya |
| P4 | M | 59 | Secondary education | Unemployed | None | 57 | 2 | 2 | 8 | SSRI(2), NDRI, SARI, **SNRI** | Yes | None | CPZ | Pla |
| P5 | F | 34 | Inc. undergraduate | Unemployed | Borderline | 9 | 25 | 8 | 14 | **SSRI**(2), **TCA**(4), SNRI(2), SARI | Yes | None | CPZ | Pla |
| P6 | F | 49 | Inc. elem. education | Unemployed | Histrionic | 41 | 8 | 3 | 3 | **SSRI**(2), SNRI | None | None | CPZ | Pla |
| P7 | F | 40 | Postgraduate | Employed | None | 38 | 2 | 2 | 6 | **SSRI**, TCA | Yes | None | CPZ | Pla |
| P8 | F | 46 | Inc. elem. education | Unemployed | Borderline | 13 | 33 | 5 | 36 | **SSRI**(4), SNRI, **TCA** | None | None | CPZ | Pla |
| P9 | M | 21 | Inc. undergraduate | Employed | None | 19 | 2 | 1 | 24 | **SSRI**, **NSSRI** | Yes | None | APZ | Pla |
| P10 | M | 56 | Inc. elem. education | Unemployed | Histrionic | 17 | 39 | 5 | 9 | **SSRI**, **TCA**, SNRI | Yes | None | DZP | Aya |
| P11 | F | 26 | Secondary education | Sick leave | Histrionic | 24 | 2 | 3 | 6 | **SNRI**, TCA, SSRI | Yes | None | CPZ | Aya |
| P12 | F | 54 | Elementary education | Unemployed | Histrionic | 40 | 14 | 4 | 12 | **SSRI**(2), TCA | Yes | None | CPZ | Aya |
| P13 | M | 56 | Inc. elem. education | Sick leave | Histrionic | 34 | 20 | 5 | 2 | **TCA**(2), SSRI | Yes | None | CPZ | - |
| P14 | F | 46 | Secondary education | Sick leave | Borderline | 29 | 17 | 4 | 12 | **SSRI**(2), SNRI | Yes | None | BZP | Pla |
| P15 | F | 47 | Elementary education | Sick leave | Histrionic | 44 | 2 | 2 | 5 | **SSRI**(2), **TCA** | None | None | CPZ | Pla |

A=ayahuasca; P=placebo; F=female; M=male; Inc=incomplete; Elem=elementary; TCA=tricyclic antidepressant; SSRI=selective serotonin-reuptake inhibitor; NDRI=noradrenaline–dopamine-reuptake inhibitor; NSSRI=noradrenaline and specific serotonin-reuptake inhibitor; SNRI=serotonin–noradrenaline reuptake inhibitor; SARI=serotonin antagonist and reuptake inhibitor; MA=melatonergic antidepressant. ECT = Electroconvulsive Therapy. ALP=alprazolam; BZP=bromazepam; CZP=clonazepam; DZP=diazepam. Current patients’ medication(s) before washout period appear in bold. *Patient A13 was not under treatment (for 6 months) at enrolment and did not remember the last medication used.

**Table S3.** HAM-D average scores and within-group effect sizes (Cohen-d) between baseline and seven days (D7) after dosing.

|  | **HAM-D** | | | |  | |
| --- | --- | --- | --- | --- | --- | --- |
|  | Baseline | | D7 | |  | |
|  | Aya | Pla | Aya | Pla |  | |
| Mean (SD) | 24.07 (5.34) | 19.73 (4.59) | 9.72 (7.39) | 16.92 (7.36) | |  |
| Cohen’s d | - | - | 2.22 | 0.46 |  | |
| 95% CI | - | - | 1.28 to 3.17 | -0.27 to 1.18 |  | |

*Aya=ayahuasca; Pla=placebo; D7=seven days after dosing.

**Table S4.** MADRS average scores and within-group effect sizes (Cohen-d) between baseline and each timepoints.

|  | **MADRS** | | | | | | | |
| --- | --- | --- | --- | --- | --- | --- | --- | --- |
|  | Baseline | | D1 | | D2 | | D7 | |
|  | Aya | Pla | Aya | Pla | Aya | Pla | Aya | Pla |
| Mean (SD) | 36.14 (6.12) | 30.13 (5.55) | 12.65 (10.27) | 21.49 (10.90) | 10.32 (10.44) | 19.09 (10.44) | 11.58 (10.27) | 26.76 (10.11) |
| Cohen’s d | - | - | 2.78 | 1.01 | 3.05 | 1.35 | 2.90 | 0.41 |
| 95% CI | - | - | 1.74 to 3.82 | 0.24 to 1.78 | 1.94 to 4.16 | 0.53 to 2.17 | 1.84 to 3.97 | -0.31 to 1.14 |

*Aya=ayahuasca; Pla=placebo; D1=one days after dosing; D2=two days after dosing; D7=seven days after dosing

**Table S5.** Acute changes were measured after 1:40h, 2:40h and 4h ayahuasca or placebo intake. For the Brief Psychiatric Rating Scale Positive subscale - BPRS+, higher values indicate increased psychotomimetic symptoms. For the Clinician-Administered Dissociative States Scale (CADSS), higher values indicate increased dissociative symptoms.

|  | **Ayahuasca** | **Placebo** | **p-value** | |
| --- | --- | --- | --- | --- |
| BPRS |  |  |  |  |
| Baseline | 1.71 (1.98) | 1.00 (1.19) | - |  |
| 1:40h | 2.08 (1.73 | 0.53 (0.74) | 0.24 |  |
| 2:40h | 1.31 (1.49) | 0.60 (1.12) | 0.61 |  |
| 4h | 0.93 (1.27) | 0.73 (1.03) | 0.62 |  |
| CADSS |  |  |  |  |
| Baseline | 15.43 (17.43) | 15.40 (18.70) | - |  |
| 1:40h | 20.80 (15.67) | 14.07 (17.20) | 0.052 |  |
| 2:40h | 18.58 (20.10) | 14.80 (20.19) | 0.73 |  |
| 4h | 15.17 (17.60) | 11.20 (17.93) | 0.64 |  |

Data are represented as mean (SEM).

**Table S6.** Adverse events during the dosing session.

|  | **Ayahuasca** | **Placebo** | **p-value** |
| --- | --- | --- | --- |
| Nausea (n, %) | 10 (71%) | 4 (26%) | 0.027 |
| Vomiting (n, %) | 8 (57%) | 0 (0%) | 0.0007 |
| Anxiety (n, %) | 7 (50%) | 11 (73%) | 0.263 |
| Restlessness (n, %) | 7 (50%) | 3 (20%) | 0.128 |
| Headache (n, %) | 6 (42%) | 8 (53%) | 0.715 |
| Diarrhea (n, %) | 1 (7%) | 0 (0%) | 0.483 |

**Table S7.** Average HRS and MEQ30 ratings for each subscale and factor assessing the psychedelic experience during the dosing session.

|  | **Ayahuasca** | **Placebo** | **p-value** |
| --- | --- | --- | --- |
| ***Hallucinogen Rating Scale*** |  |  |  |
| Perception | 25.11 (4.22) | 1.79 (1.00) | <0.0001 |
| Somaesthesia | 28.99 (3.74) | 8.52 (2.02) | <0.0001 |
| Cognition | 32.05 (2.63) | 8.48 (2.19) | <0.0001 |
| Intensity | 57.47 (6.39) | 13.45 (3.40) | <0.0001 |
| Volition | 43.51 (5.06) | 17.63 (2.92) | 0.0003 |
| Affect | 26.47 (3.52) | 22.06 (3.42) | 0.38 |
| ***Mystical Experience Questionnaire*** |  |  |  |
| Mystical | 28.67 (8.04) | 7.048 (2.17) | 0.0493 |
| Positive Mood | 41.67 (11.95) | 22.38 (8.78) | 0.32 |
| Transcendence of time and space | 50 (9.08) | 5.71 (3.96) | 0.0008 |
| Ineffability | 60 (11.13) | 10.48 (6.80) | 0.0031 |
| Total | 38.67 (5.81) | 10.19 (2.32) | 0.0037 |

All data are expressed as a percentage of maximal score. Data are represented as means (SEM).

**Figure S1. Individual HAM-D & MADRS scores at each time point.** Reddish colors mean more severe depression, while whitish mean less severity. Note how colors are whitish in the ayahuasca group when compared to the placebo, particularly at D7.

**Figure S2. Individual %-change from baseline of MADRS & HAM-D scores at each time point.** Positive responses appear in bluish, while reddish relates to negative response. All subjects in the ayahuasca group improved, while some patients in the placebo group have worsened slightly: bluish colors (positive response) are predominantly found in the ayahuasca group, when compared to the placebo, that sometimes assumes a reddish pattern (negative response).

**Figure S3. Pearson’s correlations between BPRS+ and CADSS changes at 1h40 and MADRS changes at D7 in each group separately.** The ayahuasca group is presented in red, and the placebo in blue. **a)** No significant correlations were found in ayahuasca group (r=0.45; p=0.14) or placebo (r=-0.11; p=0.69) between BPRS+ score changes (1h40 - Basal) and MADRS changes from baseline to D7. **b)** No significant correlations were found in ayahuasca group (r=0.00; p=0.99) or placebo (r=-0.26; p=0.36) between CADSS score changes (1h40 - Basal) and MADRS changes from baseline to D7.

**Figure S4.** **Individual scores for each HRS subscale.** The ayahuasca group is represented in red, and the placebo in blue. Dots represent individual scores. Bars represent mean ± SEM. We found significant differences between groups in five subscales: perception (p<0.0001), somaesthesia (p<0.0001), cognition (p<0.0001), intensity (p<0.0001), and volition (p=0.0003). Only affect was not significantly different between groups (p=0.38). Asterisks indicate significant differences between ayahuasca and placebo (**p*<0.05, ***p*<0.01, ****p*<0.001, ****p<0.0001, p-value uncorrected).

Figure S5. Individual MEQ30 scores for each factor. The ayahuasca group is represented in red, and the placebo in blue. Dots represent each individual score. Bars represent mean ± SEM. We found significant differences between groups in mystical (p=0.049), transcendence of time and space (p=0.0008) and ineffability (p=0.003) factors, and in the total MEQ30 scores (p=0.004). Only the positive mood factor (p=0.32) was not significantly different between groups. Asterisks indicate significant differences between ayahuasca and placebo (*p<0.05, **p<0.01, ***p<0.001, ****p<0.0001, p-value uncorrected).

Figure S6. Pearson’s correlations between HRS subscales and MADRS score changes at D7 in each group separately. The ayahuasca group is presented in red, and the placebo in blue. No significant correlations were found. For ayahuasca group: perception (r=0.09, p=0.78), somaesthesia (r=-0.26, p=0.39), cognition (r=-0.34, p=0.26), intensity (r=-0.12, p=0.70), volition (r=-0.54, p=0.06) and affect (r=0.29, p=0.34). For placebo group: perception (r=0.23, p=0.42), somaesthesia (r=0.31, p=0.27), cognition (r=0.08, p=0.78), intensity (r=0.13, p=0.67), volition (r=-0.07, p=0.81) and affect (r=0.34, p=0.23).

Figure S7. Pearson’s correlation between HRS subscales and MADRS score changes at D7 including only responders from the ayahuasca group. We observe significant positive correlation between perception and changes in MADRS at D7 (r=0.90, p=0.002). Correlations between MADRS score changes from baseline to D7 with the somaesthesia (r=0.58, p=0.13), cognition (r=0.60, p=0.11), intensity (r=0.08, p=0.85), volition (r=0.38, p=0.35), and affect (r=0.51, p=0.20) subscales were not significant when considering ayahuasca responders only.

**Figure S8. Pearson’s correlation between MEQ30 factors and MADRS score changes at D7 in each group separately.** We observe significant negative correlation with transcendence of time and space (r=-0.84, p=0.009) in the ayahuasca group. The remaining correlations were not statistically significant. For ayahuasca group: total MEQ30 score (r=-0.22, p=0.59), mystical (r=-0.27, p=0.51), positive mood (r=0.43, p=0.29) and ineffability (r=0.26, p=0.54). For placebo group: total MEQ30 score (r=0.66, p=0.10), mystical (r=0.49, p=0.26), positive mood (r=0.61, p=0.14) and ineffability (r=-0.09, p=0.84).

**References**

Callaway JC, et al., Quantitation of N,N-dimethyltryptamine and harmala alkaloids in human plasma after oral dosing with ayahuasca. Journal of analytical toxicology, v. 20, n. 6, p. 492–497, 1996.

Pires APS, et al., Gas chromatographic analysis of dimethyltryptamine and β-carboline alkaloids in Ayahuasca, an amazonian psychoactive plant beverage. Phytochemical Analysis, v. 20, n. 2, p. 149–153, 2009.
